# KDM4B Overexpression Promotes the Growth, Migration, and Invasion of Rheumatoid Arthritis Fibroblast-Like Synoviocytes by Activating STAT3 Pathway

**DOI:** 10.1007/s10528-021-10042-1

**Published:** 2021-04-28

**Authors:** Xin Zhang, He Nan, Jialong Guo, Jinyu Liu

**Affiliations:** 1 Department of Rheumatology and Immunology, China-Japan Union Hospital of Jilin University, 126 Xiantai Street, Changchun, 130033 Jilin China; 2 Department of Gynecologic Oncosurgery-1, Ji Lin Tumor Hospital, Changchun, 130031 Jilin China

**Keywords:** KDM4B, STAT3, Rheumatoid arthritis, Fibroblast-like synoviocytes

## Abstract

In rheumatoid arthritis (RA), fibroblast-like synoviocytes (FLS) present a unique aggressive phenotype and have a passive response to the inflammatory microenvironment, which are critical for the disease’s progression. KDM4B, as a histone demethylase, functions as an oncogenic factor in many cancers and is implicated in osteoclastogenesis as well as pro-inflammatory cytokine release in inflammatory diseases. However, the effects of KDM4B on RA FLS have not been reported. To investigate this issue, our study determined the expression of KDM4B in RA FLS using RT-qPCR and western blot. The effects of KDM4B on RA FLS viability, apoptosis, migration, and invasion were detected by MTT, flow cytometry, transwell migration, and invasion assays. Furthermore, the interaction of KDM4B with STAT3 signaling was studied by western blot, MTT, flow cytometry, transwell migration, and invasion assays. The experimental results showed that KDM4B expression was upregulated in RA synovial tissues and FLS as compared to healthy control tissues and normal FLS. Knockdown of KDM4B obviously suppressed RA FLS viability, migration and invasion, and induced apoptosis. In addition, knockdown of KDM4B in RA FLS decreased the expression of p-STAT3 and MMP-9 but increased cleaved caspase-3 expression compared with the control group. Moreover, KDM4B overexpression could promote cell growth, migration and invasion, and suppress apoptosis in RA FLS by activating STAT3 signaling. Therefore, these findings provide new insight for understanding the pathogenesis of RA and indicate that KDM4B may have a potential to be an effective therapeutic target for RA.

## Introduction

Rheumatoid arthritis (RA) is known as a chronic autoimmune disease (Safiri et al. 2019). Both environmental and genetic risk factors can cause a cascade of immune reactions and firstly lead to the hyperplasia of synovial tissue that lines the joint capsules and produces synovial fluid for polyarticular joints (Gibofsky 2012; Kumar et al. 2016). Subsequently, the inflammatory changes result in cartilage and bone destruction as well as systemic complications, which reduce life expectancy and enhance economic costs (McInnes and Schett 2011; Pisetsky 2017). Conventional and biologic disease-modifying antirheumatic drugs (DMARDs) have roles in easing symptoms of RA, and yet sometimes fail responses (Wasserman 2011). In the synovial tissue, there are two main types of synoviocytes: fibroblast-like synoviocytes (FLS) and macrophage-like synoviocytes (MLS; Hong et al. 2018). FLS, the dominant cellular component of synovial tissues, play an important role in the progression of RA (Bartok and Firestein 2010). FLS have a unique aggressive phenotype in RA and a passive response to the inflammatory microenvironment (Doody et al. 2017). RA FLS secrete inflammatory cytokines that perpetuate inflammation and produce specific matrix metalloproteinases that promote cartilage destruction (Bustamante et al. 2017). Many markers are closely associated with RA FLS, thereby affecting the progression of RA (Aletaha et al. 2010). For instance, Midkine is a key mediator for cell growth and several inflammatory conditions, which is found to be overexpressed in RA patients and RA FLS and has important diagnostic value in RA patients (Abdel Ghafar et al. 2020). PICSAR overexpression promotes cell growth and invasion of FLS to aggravate joint destruction (Bi et al. 2019). Hence, further exploring the molecular mechanisms of FLS will help understand the pathogenesis of RA and identify new therapeutic methods.

KDM4B is one of the KDM4/JMJD2 family members that are known as histone demethylases (Berry and Janknecht 2013). Like other members of the KDM4 family, KDM4B can function to demethylate di- and tri-methylated histone H3 at lysine 9 and lysine 36 (Li et al. 2020). KDM4B is frequently overexpressed in many cancers and is involved in diverse biological processes such as DNA damage, cell death, and cell-cycle arrest (Wilson and Krieg 2019). It is reported that KDM4B is associated with ERα (Estrogen receptor alpha)-dependent transcription and knockdown of KDM4B suppresses cell proliferation as well as tumor progression in breast cancer (West et al. 2016). KDM4B promotes the development of colorectal cancer through controlling cell-cycle progression and apoptosis (Li et al. 2020). KDM4B overexpression can promote the epithelial–mesenchymal transition and induce gastric cancer metastasis and proliferation (Jing et al. 2018; Zhao et al. 2013). Knocking-out KDM4B is found to increase adipogenic differentiation and reduce osteogenic differentiation in mesenchymal stem cells (MSCs; Ye et al. 2012). Nevertheless, recent study suggests that inhibition of KDM4B can reduce osteoclastogenesis and suppress pro-inflammatory cytokine release induced by bacteria in periodontal disease (Kirkpatrick et al. 2018). Since periodontal disease is a chronic inflammatory disease leading to the teeth damage (Kirkpatrick et al. 2018), we speculate that KDM4B may have important roles in inflammatory diseases. However, the effects of KDM4B on RA have not been reported.

Signal transducer and activator of transcription (STAT) 3, a member of STAT family of transcription factors, functions to translocate extracellular signals from a cellular receptor to the nucleus and regulates the transcription of specific genes, thereby controlling many cellular biological processes including cell survival, apoptosis, proliferation, and immunity (Guanizo et al. 2018; You et al. 2015). Accumulating evidence suggests that dysregulated STAT3 is closely associated with oncogenesis and cell transformation (Sgrignani et al. 2018). Furthermore, STAT3 is proved to be a potential biomarker target for RA therapy because STAT3 can regulate inflammatory signals and osteoclastogenesis (Oike et al. 2017). Moreover, loss of KDM4B promotes DNA damage through inhibiting the activity of STAT3 signaling pathway in colorectal cancer (Chen et al. 2014; Deng et al. 2018), and stimulation with IL-6/sIL-6R suppresses H3K9me3 through the recruitment of KDM4B at the STAT-binding site in RUNX2 promoter region to induce differentiation of human vascular smooth muscle cells into osteoblast-like cells (Kurozumi et al. 2019). Hence, we speculated that KDM4B might regulate the activity of STAT3 signaling to affect the development of RA, and yet the interaction of KDM4B with STAT3 in RA has not been studied.

The aim of the present study was to investigate the mechanisms of KDM4B and the interaction between KDM4B and STAT3 in RA. We used RT-qPCR and western blot to detect the expression of KDM4B in RA synovial tissues and FLS, and determined RA FLS viability, migration, invasion, and apoptosis to explore the interaction of KDM4B with STAT3 in RA.

## Materials and Methods

### Human Synovial Tissue Samples

Synovial tissue samples were collected from 45 patients with RA who met the American College of Rheumatology criteria (Cohen and Emery 2010) and underwent synovectomy or total knee arthroplasty in China–Japan Union Hospital of Jilin University. Besides, 30 normal control synovial tissues were obtained from individuals who underwent arthroscopic surgery for traumatic joint damage and who had no history of chronic or acute arthritis. All participants in this study signed informed consent. This experiment got approval from the Ethics Committee of China–Japan Union Hospital of Jilin University.

### Isolation and Culture of Primary FLS

FLS were isolated from synovial tissues of RA patients and joint trauma patients as previously described (Bi et al. 2019; Rosengren et al. 2007). The tissue samples were cut into small pieces (1–2 mm^3^), digested with 1 mg/ml collagenase in DMEM (Dulbecco’s Modified Eagle Medium, Gibco, Carlsbad, CA, USA) containing 1% penicillin/streptomycin at 37℃ in a shaking incubator to isolate synoviocytes. The cells were seeded into cell culture dishes in DMEM supplemented with 1% penicillin/streptomycin and 10% FBS (fetal bovine serum, Gibco) at 37℃ under 5% CO_2_. When confluence reaches 90–100%, cells were trypsinized for subculture. FLS were identified by flow cytometry staining with CD68 FITC and CD90 PE monoclonal antibodies. FLS passaged 4–8 times were used for further analysis.

### Cell Transfection and Treatment

shRNAs targeting KDM4B (shKDM4B-1#, shKDM4B-2#) and negative control (shNC) were purchased from GenePharma (Shanghai, China). KDM4B overexpression plasmid and negative control plasmid (NC) were constructed by Hanbio (Shanghai, China). The above vectors were, respectively, transfected into RA FLS using Lipofectamine 3000 (Thermo Fisher Scientific, Waltham, MA, USA). Then, the transfected FLS were cultured in DMEM containing 1% penicillin/streptomycin and 10% FBS for the following experiments. For the inhibition of STAT3 activity, the transfected FLS, stably expressing KDM4B, were treated with Stattic (10 μM, Sigma) for 24 h, which were prepared for the following experiments.

### Real-Time Quantitative PCR (RT-qPCR)

Total RNA from synovial tissues and FLS was extracted using TRIzol reagent (Thermo Fisher Scientific), quantified by NanoDrop™ One/One^C^ (Thermo Fisher Scientific) and reverse transcribed to cDNA using high-capacity cDNA Reverse Transcription Kit (Thermo Fisher Scientific). The obtained cDNA as a template was applied for real-time PCR using Power SYBR™ Green PCR Master Mix (Thermo Fisher Scientific). The cDNAs were amplified as the following protocol: an initial denaturation step at 94 °C for 10 min, followed by 35 cycles of denaturation at 94 °C for 45 s, annealing at 50 °C for 45 s, and synthesis at 72 °C for 30 s. Amplification product length of KDM4B primers was 78 bp and that of β-actin primers was 150 bp. Besides, an external standard curve for KDM4B sequence was prepared to obtain the amplification efficiency (En = 0.95), and β-actin had the same amplification efficiency. Relative expression of KDM4B was calculated using the $2^{{ - \Delta \Delta C_{{\text{T}}} }}$ method and normalized to the levels of the internal control, β-actin. The primers were listed as following: β-actin (Forward: 5′-CCC ATC TAT GAG GGT TAC GC -3′; Reverse: 5′-TTT AAT GTC ACG CAC GAT TTC-3′). KDM4B (Forward: 5′-GGA CTG ACG GCA ACC TCT AC-3′; Reverse: 5′-CGT CCT CAA ACT CCA CCT G-3′).

### Western Blot

Synovial tissues and FLS were lysed using RIPA lysis buffer to obtain total protein. The proteins were quantified by bicinchoninic acid assay (BCA) kit (Pierce), equally subjected to SDS-PAGE (sodium dodecyl sulfate-polyacrylamide gel electrophoresis) gel and transferred to PVDF (polyvinylidene fluoride or polyvinylidene difluoride) membrane. Next, the membranes were sealed by 5% skim milk for 1 h and incubated with primary antibodies at 4℃ overnight. After washing with TBST (Tris-buffered saline, 0.1% Tween 20) buffer, the membranes were subsequently cultured with secondary antibodies [Goat Anti-Rabbit IgG H&L (HRP), Cat no. ab6721, dilution 1/2000, Abcam] at room temperature for 1 h. Finally, the protein blots were visualized using ECL (electrogenerated chemiluminescence) solution and quantified by ImageJ software. Primary antibodies were listed as follows: KDM4B (Cat no. #8639, dilution 1/1000), β-actin (Cat no. #4970, dilution 1/1000, Cell Signaling Technology), p-STAT3 (Cat no. #52075, dilution 1/1000, Cell Signaling Technology), STAT3 (Cat no. #30835, dilution 1/1000, Cell Signaling Technology), Cleaved caspase-3 (Cat no. ab2302, dilution 1/500, Abcam), and MMP-9 (Cat no. ab228402, dilution 1/1000, Abcam) antibodies.

### Cell Viability Assay

The viability of RA FLS was detected by 3-(4,5-dimethylthiazol-2-yl)-2,5-diphenyltetrazolium bromide (MTT) assay. 48 h post-transfection, 100 μl of RA FLS suspension was seeded in 96-well plates at the density of 5 × 10^4^ cells per well and maintained in a humidified incubator at 37℃ for 24 h. Then, MTT solution (5 g/l, 20 μl) was added into each well of the plates. The cells were incubated for another 4 h. After discarding the supernatant, we added DMSO (dimethylsulfoxide, 150 μl) in the cells. The sample absorbance was measured using a microplate reader at 570 nm.

### Flow Cytometry

48 h post-transfection, RA FLS were harvested, washed with cold PBS, and suspended in 1 ml 1 × Annexin V binding buffer. After centrifugation, the supernatant was discarded, and the cells were resuspended in 500 μl binding buffer and 5 μl Annexin V-FITC (Abcam) and 5 μl propidium iodide (PI). After the cells were incubated in the dark for 5 min at room temperature, the apoptosis of RA FLS was measured using flow cytometry (Beckman Coulter, CA, USA).

### Transwell Migration and Invasion Assays

Transwell chambers with 8-μm pores (Corning, USA) were prepared with or without Matrigel (BD Biosciences) to assess the invasion and migration of RA FLS, respectively. 48 h post-transfection, RA FLS were collected, suspended in serum-free DMEM, and seeded in the upper chamber. The lower chamber was filled with DMEM supplemented with 10% FBS and 1% penicillin/streptomycin. The cells were incubated at 37℃, 5% CO_2_ for 48 h, and the cells that remained in the upper chamber were removed gently by cotton swabs. The cells that migrated or invaded in the lower membrane were fixed with 4% paraformaldehyde for 20 min and stained with 0.1% crystal violet for 10 min. The cells were visualized and counted by an inverted microscope in five random fields.

### Statistical Analysis

All assays were repeated three times in this study. The experimental data were expressed as mean ± standard deviation (SD) and analyzed by GraphPad prism 7 software. The differences between two groups were evaluated using Student’s *t* test, and multiple comparisons were assessed by one-way analysis of variance with Bonferroni’s multiple comparisons test. The difference is regarded statistically significant when a *p* value is less than 0.05.

## Results

### KDM4B Expression is Upregulated in the Synovial Tissue and FLS of RA Patients

**Fig. 1:**
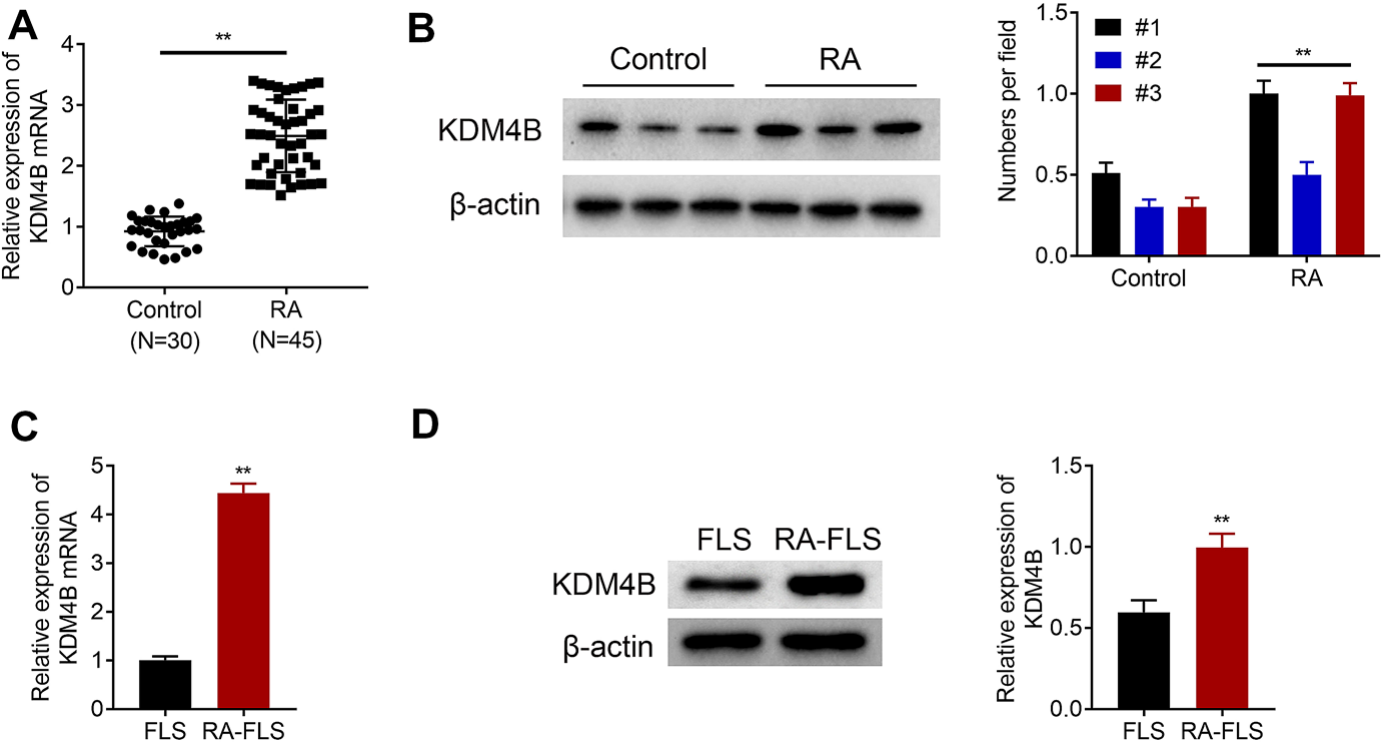
KDM4B expression is upregulated in the synovial tissue and fibroblast-like synoviocytes (FLS) of rheumatoid arthritis (RA) patients. **a** The mRNA expression levels of KDM4B in RA synovial tissues (*n* = 45) and healthy control synovial tissues (*n* = 30) were measured by RT-qPCR. **b** The protein expression of KDM4B in randomly selected RA synovial tissues (*n* = 3) and healthy control synovial tissues (*n* = 3) was examined by western blot. **c** and **d** The mRNA and protein expression levels of KDM4B in RA FLS and normal FLS were determined by RT-qPCR and western blot, respectively. **p < 0.01 versus healthy control synovial tissues or normal FLS

RT-qPCR and western blot were used to analyze the difference in KDM4B expression between RA synovial tissues and healthy control. As shown in Fig. 1a, the mRNA expression of KDM4B was significantly upregulated in RA synovial tissues (*n* = 45) compared with healthy control tissues (*n* = 30). Figure 1b indicates that the protein expression of KDM4B in randomly selected RA synovial tissues (*n* = 3) was higher than that in healthy control (*n* = 3). Furthermore, RA FLS and normal FLS were separately isolated from RA synovial tissues and healthy synovial tissues. RT-qPCR and western blot further confirmed that the mRNA and protein expression of KDM4B were upregulated in RA FLS compared with normal FLS (Fig. 1c, d).

### Knockdown of KDM4B Inhibits RA FLS Viability But Promotes Apoptosis

**Fig. 2:**
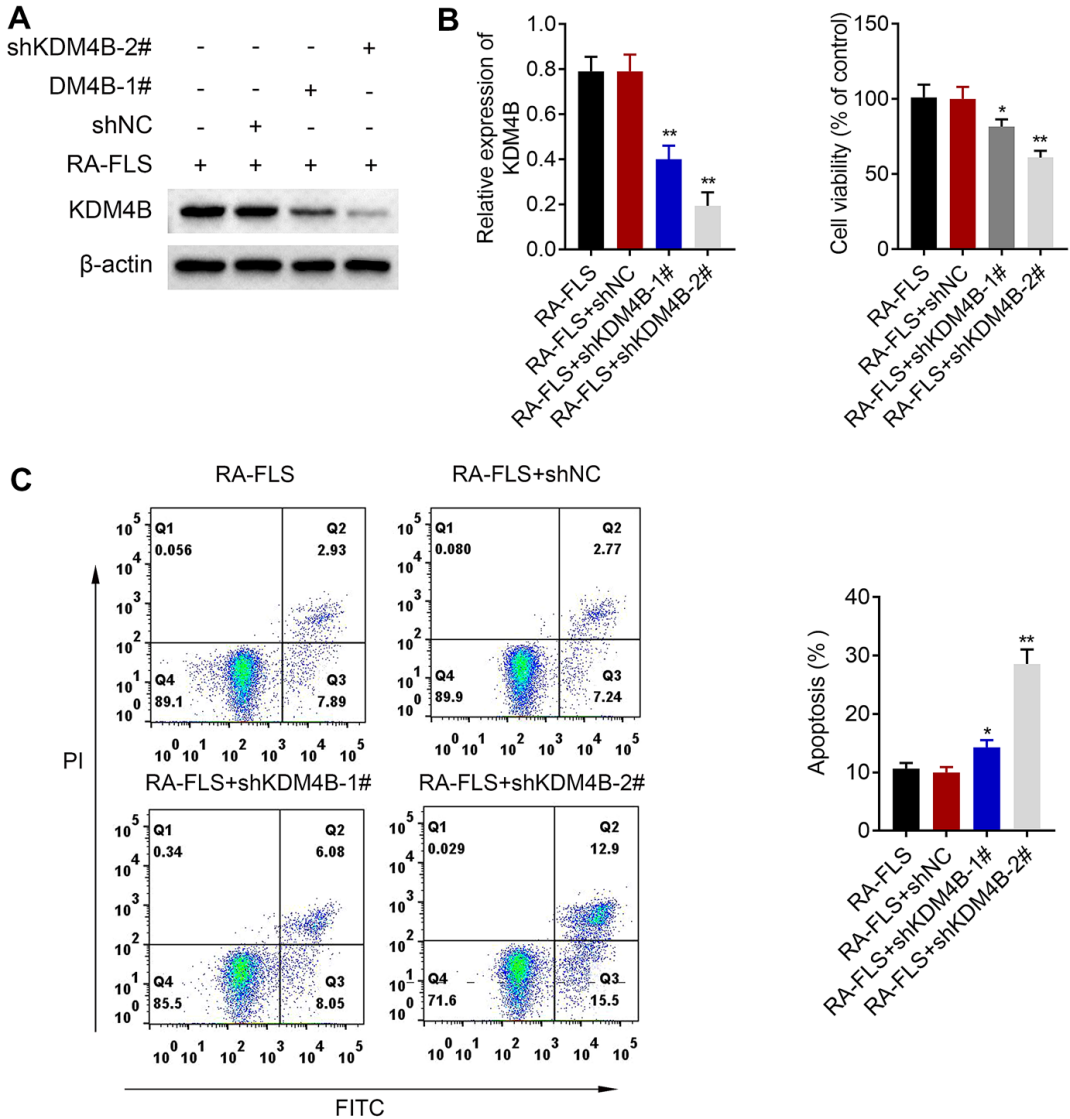
Knockdown of KDM4B inhibits RA FLS viability but promotes apoptosis. After RA FLS were, respectively, transfected with shKDM4B-1#, shKDM4B-2#, and shNC, **a** the protein expression of KDM4B was detected by western blot. **b** The viability of RA FLS was measured by MTT assay. **c** The apoptosis of RA FLS was evaluated using flow cytometry. + represents the existence of materials, but − represents the inexistence of materials. *p < 0.05, **p < 0.01 versus RA FLS + shNC

To investigate the effect of KDM4B on RA FLS survival, we carried out western blot, MTT, and flow cytometry assays. After RA FLS were, respectively, transfected with shKDM4B-1#, shKDM4B-2#, and shNC, western blot showed that the protein expression of KDM4B between shNC transfection and blank control groups had no significant difference, but shKDM4B-1# and shKDM4B-2# obviously suppressed the expression of KDM4B as compared to shNC group (Fig. 2a). MTT indicated that the viability of RA FLS was inhibited by knockdown of KDM4B compared with the shNC group (Fig. 2b). Additionally, knockdown of KDM4B considerably promoted the apoptosis of RA FLS when compared with the shNC group (Fig. 2c). Hence, knockdown of KDM4B suppressed RA FLS viability but promoted apoptosis.

### Knockdown of KDM4B Inhibits the Migration and Invasion of RA FLS

**Fig. 3:**
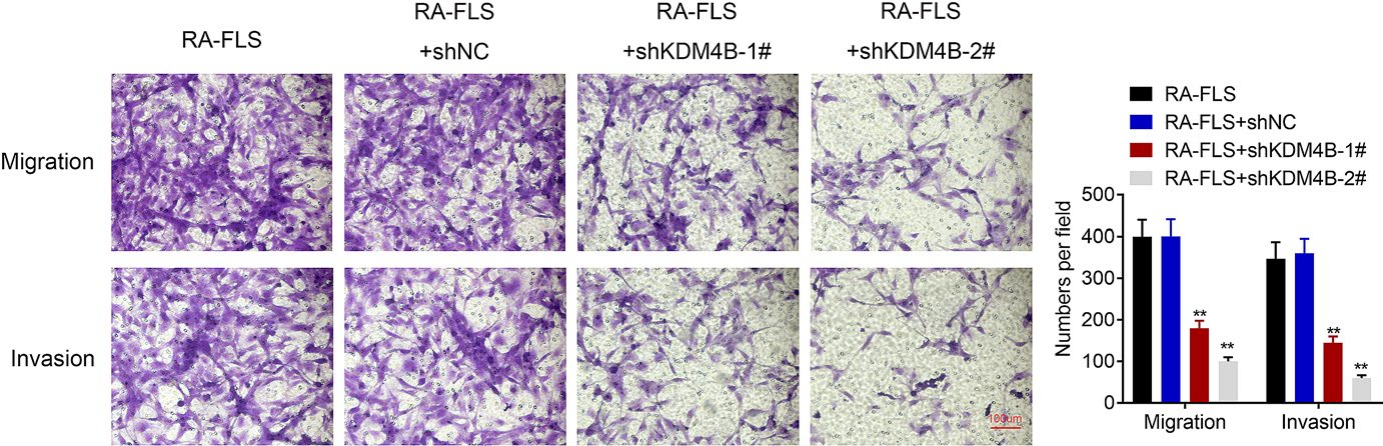
Knockdown of KDM4B inhibits the migration and invasion of RA FLS. The migration and invasion of RA FLS were, respectively, assessed by transwell migration and invasion assays. **p < 0.01 versus RA FLS + shNC

The effect of KDM4B on RA FLS metastasis was determined by transwell assays. As shown in Fig. 3, there was no significant difference in cell migrated numbers between shNC transfection and blank control groups, whereas the transfection of shKDM4B-1# and shKDM4B-2# inhibited the number of migrated RA FLS when compared with the shNC group. Meanwhile, the invaded RA FLS numbers were suppressed by the knockdown of KDM4B (Fig. 3). Thus, knockdown of KDM4B inhibited the migration and invasion of RA FLS.

### KDM4B Regulates the Activity of STAT3 Signaling in RA FLS

**Fig. 4:**
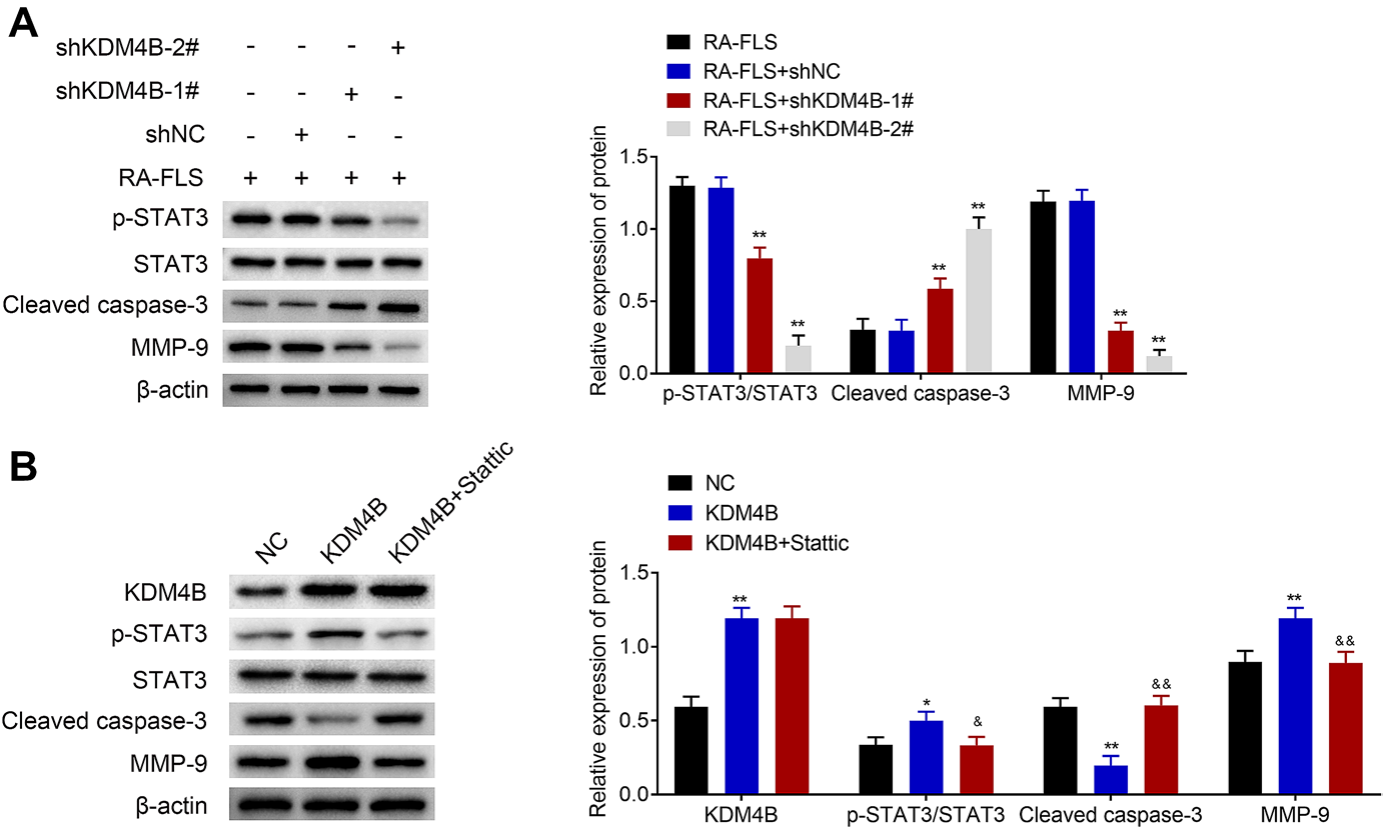
KDM4B regulates the activity of STAT3 signaling in RA FLS. **a** The expression levels of p-STAT3, STAT3, cleaved caspase-3, and MMP-9 were determined by western blot after RA FLS transfected with shKDM4B-1#, shKDM4B-2#, or shNC. **b** After KDM4B plasmid combined with or without Stattic was used to treat RA FLS, the expression levels of KDM4B, p-STAT3, STAT3, cleaved caspase-3, and MMP-9 were examined by western blot. + represents the existence of materials but − represents the inexistence of materials. *p < 0.05, **p < 0.01 versus RA FLS + shNC or NC (control plasmid); ^&^*p* < 0.05, ^&&^*p* < 0.01 versus KDM4B plasmid

Previous reports suggest that KDM4B silencing inhibits the activity of STAT3 signaling in colorectal cancer (Chen et al. 2014; Deng et al. 2018), and thus, we tried to detect whether KDM4B can regulate the activity of STAT3 signaling in RA FLS. First, the expression levels of proteins (p-STAT3, STAT3, Cleaved caspase-3, and MMP-9) in RA FLS transfected with shKDM4B-1#, shKDM4B-2#, or shNC were measured by western blot. The results showed that knockdown of KDM4B increased the level of cleaved caspase-3 and decreased the expression of p-STAT3 and MMP-9 in comparison to the shNC group (Fig. 4a). The expression changes of an apoptotic biomarker cleaved caspase-3 and an extracellular matrix-degrading enzyme MMP-9 confirmed that knockdown of KDM4B could inhibit RA FLS migration and invasion and induce apoptosis.

Second, we used Stattic, an inhibitor of STAT3 activation, to further study the interaction of KDM4B with STAT3 in RA FLS. KDM4B plasmid combined with or without Stattic was used to treat RA FLS, and the protein expression in the treated RA FLS was also detected via western blot. In Fig. 4b, KDM4B plasmid upregulated the expression of KDM4B, p-STAT3, and MMP-9 and downregulated the level of cleaved caspase-3 when compared with the control. As Stattic can potently inhibit STAT3 activation (Schust et al. 2006), and consequently the introduction of Stattic greatly downregulated the level of p-STAT3. Meanwhile, the introduction of Stattic did not change the expression of KDM4B but upregulated Cleaved caspase-3 expression and downregulated the level of MMP-9 as compared to KDM4B plasmid group. Therefore, KDM4B could regulate the activity of STAT3 signaling in RA FLS.

### KDM4B Promotes the Growth of RA FLS Through Activating the STAT3 Signaling

**Fig. 5:**
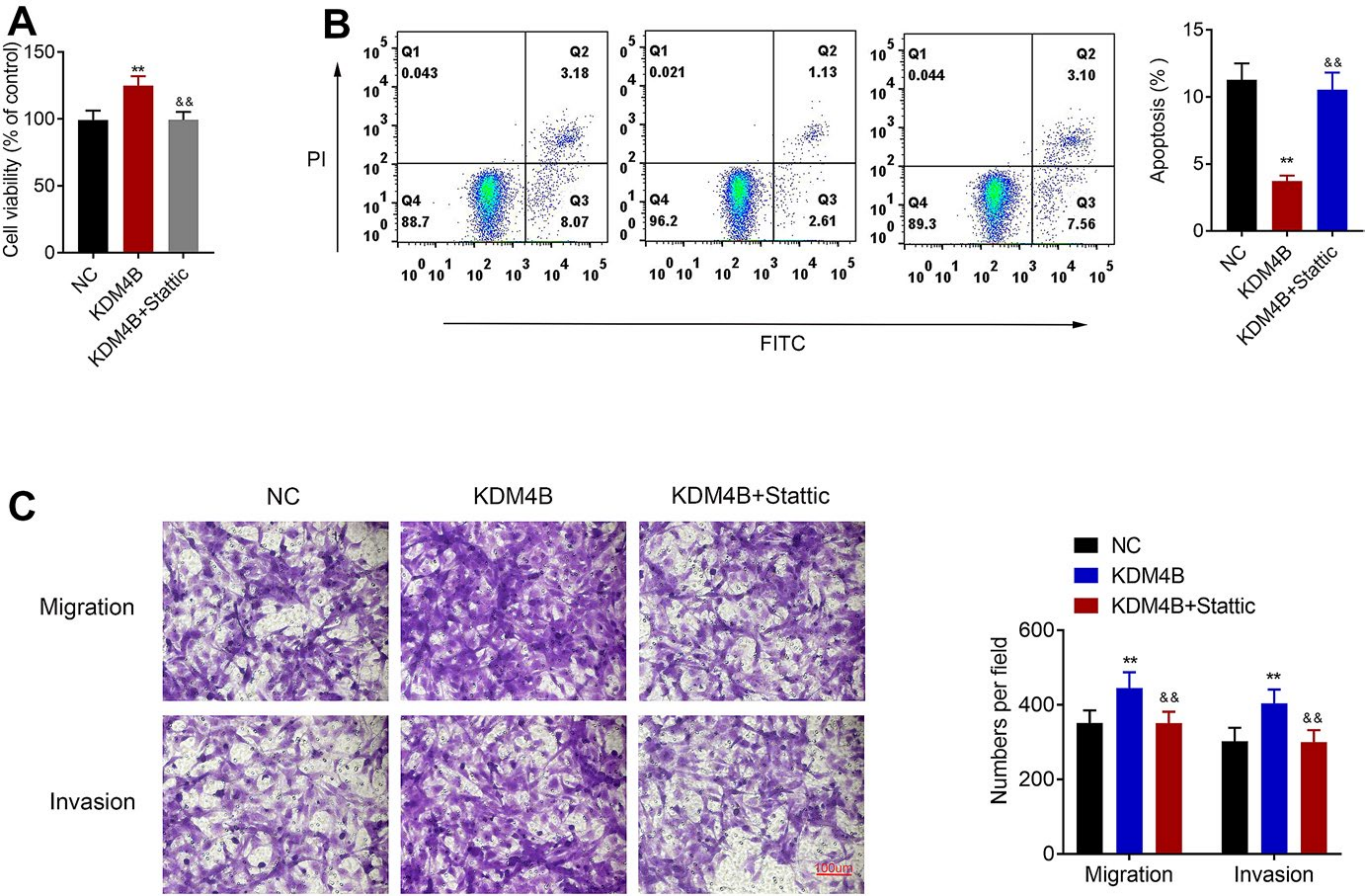
KDM4B promotes the growth of RA FLS through activating the STAT3 signaling. After KDM4B plasmid combined with or without Stattic was applied to treat RA FLS, **a** MTT was used to detect the viability of RA FLS, **b** flow cytometry was applied to measure the cell apoptosis, and **c** transwell migration and invasion assays were utilized to assess the cell migration and invasion. **p < 0.01 versus NC (control plasmid); ^&&^*p* < 0.01 versus KDM4B plasmid

MTT, flow cytometry, and transwell assays were utilized to study the interaction of KDM4B with STAT3 signaling in RA FLS. The viability of RA FLS was enhanced by KDM4B overexpression plasmid, which was reversed after the introduction of Stattic (Fig. 5a). KDM4B overexpression decreased the apoptosis of RA FLS compared with the control, while the introduction of Stattic promoted the cell apoptosis as compared to KDM4B plasmid (Fig. 5b). In addition, the increase of RA FLS migration and invasion induced by KDM4B overexpression was reversed by the introduction of Stattic (Fig. 5c). Hence, KDM4B could promote RA FLS viability, migration and invasion, and suppress apoptosis through activating STAT3 signaling.

## Discussion

In RA, activation of FLS can migrate and invade the cartilage and bone and secrete various pro-inflammatory cytokines (e.g., TNF-α, IL-1β, IL-6) and proteins associated with matrix degradation and innate immunity, which has critical roles in promoting the progression of RA (Bustamante et al. 2017). Hence, the study of the mechanisms of FLS in RA may be a potential strategy to prevent the disease’s progress. KDM4B, as a histone demethylase, is involved in many biological processes such as cell cycle arrest, proliferation, and DNA damage (Wilson and Krieg 2019). KDM4B overexpression is usually found in various cancers including breast cancer, colorectal cancer, and gastric cancer (Jing et al. 2018; Li et al. 2020; West et al. 2016; Zhao et al. 2013). Moreover, KDM4B is relevant with osteogenic differentiation in MSCs as well as osteoclastogenesis in periodontal disease (Kirkpatrick et al. 2018; Ye et al. 2012). Additionally, KDM4B silencing can inhibit pro-inflammatory cytokine release induced by bacteria in periodontal disease (Kirkpatrick et al. 2018). Thus, we speculate that KDM4B may have important roles in inflammatory diseases, and our study tried to explore the roles of KDM4B in RA. In the present study, we determined the expression of KDM4B in RA and found the overexpression in RA synovial tissues and FLS as compared to healthy control tissues and normal FLS. Furthermore, knockdown of KDM4B suppressed the viability of RA FLS but induced apoptosis. In addition, knockdown of KDM4B inhibited the migration and invasion of RA FLS. Therefore, KDM4B may be a potential biomarker for the treatment of RA through preventing the growth of RA FLS.

STAT3 is a key intracellular transcription factor that controls many cellular processes, mainly including cell differentiation, inflammation, apoptosis, and proliferation (Guanizo et al. 2018; You et al. 2015). Aberrant activation of STAT3 signaling is observed in numerous cancers and promotes the development and progression of cancers (Sgrignani et al. 2018). Accumulating evidence suggests that STAT3 activation promotes the proliferation of RA FLS and suppresses apoptosis (Chang et al. 2019; Liu et al. 2017). Moreover, loss of KDM4B inhibits the activity of STAT3 to suppress the progression of colorectal cancer (Chen et al. 2014; Deng et al. 2018), and KDM4B regulates the differentiation of vascular smooth muscle cells into osteoblast-like cells through interacting with STAT3 (Kurozumi et al. 2019). Hence, we attempted to investigate whether KDM4B can mediate the activity of STAT3 signaling in RA FLS. Our study found that knockdown of KDM4B in RA FLS significantly decreased the expression of p-STAT3 and MMP-9 but increased cleaved caspase-3 expression compared with the control group. Besides, the increase of p-STAT3 and MMP-9 expression and the decrease of cleaved caspase-3 expression induced by KDM4B overexpression were reversed by the introduction of Stattic that can potently inhibit STAT3 activation (Schust et al. 2006). Hence, KDM4B could control the activity of STAT3 signaling in RA FLS.

In addition to STAT3 responsible for cellular processes, the changes of cleaved caspase-3 and MMP-9 are critical for the biological processes of cells. Caspase-3 can be cleaved and activated via both caspase-9 and caspase-8 initiator caspases to trigger apoptosis (Shalini et al. 2015; Zhu et al. 2020). Previous reports show that the inhibition of STAT3 signaling increases the activation of caspase-9, caspase-3, and caspase-7 (Sun et al. 2017). MMP-9 is a class of matrix metalloproteinases associated with the degradation of the extracellular matrix and promotes cell migration, invasion, and angiogenesis (Huang 2018; Lin et al. 2019). STAT3 inhibition is found to decrease the expression of MMP-9 (Oh et al. 2018). Consistent with previous reports, our study found that the decrease of p-STAT3 expression caused by knockdown of KDM4B was accompanied by the decrease of MMP-9 and the increase of cleaved caspase-3. Furthermore, our study indicated that KDM4B overexpression enhanced RA FLS viability, inhibited apoptosis, and promoted cell migration and invasion, which was changed by the introduction of Stattic. Therefore, KDM4B overexpression could promote cell growth, migration, and invasion, and suppress apoptosis in RA FLS by activating STAT3 signaling.

Taken together, our study suggested that KDM4B expression was upregulated in RA synovial tissues and FLS. In addition, the aberrant high level of KDM4B in RA promoted FLS growth, migration, and invasion and inhibited apoptosis through the activation of STAT3 signaling. These results show that KDM4B may be a potential biomarker for the treatment of RA patients and provide new insight for understanding the pathogenesis of RA.

## Data Availability

All data generated or analyzed during this study are included in this published article.

## Code Availability

Not applicable.
